# Draft Genome Sequences of Two Polycyclic Tetramate Macrolactam Producers, *Streptomyces* sp. Strains JV180 and SP18CM02

**DOI:** 10.1128/MRA.01066-20

**Published:** 2020-12-10

**Authors:** Yunci Qi, Keshav K. Nepal, Jennifer Greif, Cole Martini, Chad Tomlinson, Christopher Markovic, Catrina Fronick, Joshua A. V. Blodgett

**Affiliations:** a Department of Biology, Washington University in St. Louis, St. Louis, Missouri, USA; b McDonnell Genome Institute, Washington University School of Medicine, St. Louis, Missouri, USA; Portland State University

## Abstract

Here, we report the draft genome sequences of two related *Streptomyces* sp. strains, JV180 and SP18CM02. Despite their isolation from soils in Connecticut and Missouri (USA), respectively, they are strikingly similar in gene content. Both belong to the Streptomyces griseus clade and harbor several secondary metabolite biosynthetic gene clusters.

## ANNOUNCEMENT

*Streptomyces* species produce many diverse secondary metabolites that are medicinally relevant (1). Recent advancements in genome sequencing have revealed *Streptomyces* as a reservoir of diverse biosynthetic gene clusters (BGCs). A high proportion of *Streptomyces* harbor BGCs encoding polycyclic tetramate macrolactams (PTMs), molecules of interest due to their structural complexity, atypical biosyntheses, and biomedical significance (2–4). *Streptomyces* sp. strain JV180 and *Streptomyces* sp. strain SP18CM02 are soil isolates found to carry PTM BGCs. The isolation of strain JV180 was previously reported (3), while strain SP18CM02 was recently isolated using identical methods from calcium carbonate-treated soil collected at Tyson Research Center in Eureka, MO, using low-tryptone-yeast extract (LTY) medium supplemented with nalidixic acid.

Both strains were grown in Trypticase soy broth supplemented with 0.6% glycine at 28°C, and genomic DNA was extracted with the Qiagen DNeasy UltraClear microbial kit for Illumina sequencing or phenol-chloroform (5) for PacBio sequencing. For PacBio sequencing, genomic DNA was sheared to approximately 15 kb using Covaris g-TUBEs. The PacBio SMRTbell Express template prep kit 2.0, barcoded overhang adapter kit 8A, and barcoded overhang adapter kit 8B were used for library construction. Final library pools were assessed on an Agilent 2100 Bioanalyzer using a DNA 12000 kit. DNA polymerase binding complex was processed using the PacBio Sequel binding kit 3.0 and sequencing primer 4. The PacBio Sequel sequencing plate 3.0 and a single-molecule real-time (SMRT) cell (1M v3 Tray) were used for the sequencing run. The resulting reads were evaluated based on mean read length, which was 27,869 bp. PacBio read error correction, adapter trimming, and assembly were done in Hierarchical Genome Assembly Process 4 (HGAP4). Default parameters were used with the exception of the advanced parameter, “aggressive mode.” The assembly was polished in Pilon 1.23 with Illumina reads, which were obtained as described below (6, 7).

For Illumina sequencing, dual-indexed libraries were constructed with 2 μg of genomic DNA (gDNA) utilizing the TruSeq PCR-free library prep kit (Illumina) on the SciClone next-generation sequencing (NGS) instrument (Perkin Elmer) targeting 550-bp inserts. Then, 50 μl of gDNA was fragmented on an LE200 Covaris instrument, and the library was evaluated on a LabChip GX system (Perkin Elmer). The library was sequenced on a NovaSeq 6000 (Illumina) S4 300 cycle flow cell for 2 × 150-bp paired-end reads, and NovaSeq Real Time Analysis 3.3.3 software (Illumina) was used to ensure that ≥80% of resulting bases had a quality score of ≥Q30. FLEXBAR 3.4 (8) was used for adapter trimming, and MEGAHIT (9) was used for genome assembly with default parameters. Genomic features were annotated with Rapid Annotations using Subsystems Technology 2.0 (10) and antiSMASH (11) (Table 1).

**TABLE 1:** Genomic features of *Streptomyces* sp. strains JV180 and SP18CM02

*Streptomyces* sp. strain	Sequencing method	No. of reads	Sequence size (bp)	Genome size (bp)	Fold coverage (×)	No. of contigs	*N*_50_ (bp)	G+C content (%)	No. of CDS^*a*^	No. of RNAs
JV180	PacBio	920,865	2,597,107,056	8,006,517	324.37	5	7,244,875	72.6	7,353	83
	Illumina	22,533,770	3,402,599,270							
SP18CM02	Illumina	16,938,420	2,557,701,420	8,024,422	318.74	344	431,047	72.7	7,374	76

a CDS, coding DNA sequences.

Strains JV180 and SP18CM02 share 100% identical 16S rRNA sequences, and their closest match in GenBank was Streptomyces californicus strain NRRL B-3320 (99.93%), a strain that has a similar genome size (8,023,053 bp) and belongs to the Streptomyces griseus clade (12). MUSCLE pairwise alignments (13) revealed that strains JV180 and SP18CM02 share 99.93, 99.76, 99.29, 99.91, and 99.53% nucleotide identities for *atpD*, *gyrB*, *recA*, *rpoB*, and *trpB*, respectively. Strains JV180 and SP18CM02 possess 34 and 35 BGCs, respectively, and all strain JV180 BGCs are conserved in strain SP18CM02.

### Data availability.

The draft genome sequences of strains JV180 and SP18CM02 were deposited in DDBJ/ENA/GenBank under accession numbers JACGMP000000000 and JACGMQ000000000, respectively. The SRA accession numbers for strain JV180 are SRR12430886 and SRR12430887 for Illumina and PacBio sequencing, respectively, and that for strain SP18CM02 is SRR12430885. The versions described in this paper are the first versions.
